# Determinants of Purchase Intention for Meat-Based Chilled Ready Meals in New Zealand: A Consumer Behaviour Perspective

**DOI:** 10.3390/foods14061038

**Published:** 2025-03-18

**Authors:** Chathurika S. S. Samarakoon Mudiyanselage, Mustafa M. Farouk, Carolina E. Realini, Kevin Kantono, Nazimah Hamid

**Affiliations:** 1 AgResearch Limited, Ruakura Research Centre, 10 Bisley Road, Hamilton 3214, New Zealand; 2 Centre for Future Foods, Auckland University of Technology, Private Bag 92006, Auckland 1142, New Zealand; 3 AgResearch Limited, Te Ohu Rangahau Kai, Massey University Campus, University Ave., Palmerston North 4474, New Zealand

**Keywords:** purchase intention, consumer choice, meat-based, chilled ready meals, consumer behaviour

## Abstract

The chilled ready meal market in New Zealand has witnessed substantial growth, fuelled by the increasing demand for convenient food options. This study integrates the theory of consumption value (TCV) with the theory of planned behaviour (TPB) to explore how lifestyle, consumer knowledge, sensory properties, subjective norms, consumer choice behaviour, consumption habits, and perceived behavioural control influence the purchase intention of meat-based chilled ready meals. Partial least squares path modelling (PLSPM) was used to verify the conceptual model using data from an online survey of 464 meat-based ready meal consumers from New Zealand. The results revealed that consumer attitudes towards meat-based chilled ready meals were positively influenced by lifestyle, consumer knowledge, and sensory properties, but these attitudes negatively impacted purchase intention, emphasising the complexity of consumer decision-making. Subjective norms, particularly social influences, significantly shaped consumers’ intentions. Furthermore, consumer choice behaviour, including conditional value, epistemic value, and emotional value, significantly influenced purchase intentions, with consumption habits emerging as the strongest predictor. Price had the highest influence on perceived behavioural control, while packaging information had little direct effect on perceived behavioural control. The findings of this study provide actionable insights for businesses to tailor marketing strategies and enhance consumer acceptance by improving product quality and sensory appeal of meat-based chilled ready meals in New Zealand.

## 1. Introduction

The global ready meal market has undergone significant expansion in recent years, primarily driven by increased demand for convenience foods [1]. It is projected to grow from NZD 156.33 billion in 2022 to NZD 248.13 billion by 2029, with a compound annual growth rate (CAGR) of 6.8% from 2022–2029 [2]. This global trend has led to an expansion in the availability and variety of pre-prepared meal solutions, including chilled ready meals, offered by the food industry and retailers. Additionally, ready meal online subscriptions and delivery services are becoming increasingly popular in New Zealand, allowing consumers to easily order ready meals on a weekly basis for their entire family. According to StatsNZ, the weekly expenditure of a family on ready-to-eat food including ready meals has experienced notable growth in recent years, while spending on grocery items has decreased [3,4]. This suggests the importance of gaining a deeper understanding of the factors influencing consumers’ intentions to purchase ready meals.

A ready meal can be defined as a complete meal that requires few or no extra ingredients, prepared by external providers and designed to fully and speedily substitute the main course of a homemade main meal [5,6,7]. Ready meals differ from ready-to-eat (RTE) takeaway foods, as the former still require some cooking or re-heating prior to serving [8]. Consumers are increasingly gravitating towards healthy and high-quality ready meals that are prepared fresh with minimal or no preservatives, often replacing home-cooked meals [9]. Despite the increasing availability and demand for these products, the key factors influencing and driving consumers’ intentions to purchase chilled ready meals in New Zealand are relatively unknown.

While several studies have examined the determinants of ready meal purchase intention in general, there is limited research focused specifically on meat-based chilled ready meals, which represent a significant segment of the ready meal market. Existing studies have identified several factors influencing consumer’s intention to purchase ready meals and convenience foods, including socio-demographic and economic determinants such as household size, age, income level, and employment status [1,10,11]. Other factors include consumption habits [6], moral attitude [12], health [1,5], and personal values [13]. According to Olsen and Sijtsema [12], factors like working families, long work hours, and commuting time, the desire to spend less time on meal preparation, and to maximise leisure time have led to consumer demands for convenience foods like takeaways and ready meals. The lack of culinary skills and experience with preparing meals at home have boosted the demand for easy-to-prepare meals [14,15]. However, these studies have not sufficiently addressed the specific consumer motivations behind the purchase of meat-based chilled ready meals, which may differ from other chilled ready meal categories due to distinct consumer preferences for meat products. It is important to explore this gap because meat-based chilled ready meals present unique challenges and opportunities within the broader ready meal market. Factors such as sensory quality including freshness, perception of healthiness, and convenience may play a critical role in shaping consumer preferences for this product category. To date, there is no comprehensive study that has investigated the underlying factors influencing the purchase intention of meat-based chilled ready meals specifically, particularly within the context of New Zealand.

The aim of this study is, therefore, to investigate the determinants of purchase intention of meat-based chilled ready meals in New Zealand using human behavioural research methodologies. These methodologies are frequently employed in consumer studies to ascertain the underlying factors determining food-related behaviours. By applying a social psychological approach that integrates the theory of planned behaviour (TPB) with the theory of consumption value (TCV), this study seeks to identify the key factors influencing consumers’ intentions to purchase meat-based chilled ready meals in New Zealand. This research will also contribute to the existing literature by addressing the specific gap related to meat-based chilled ready meals.

## 2. Theoretical Background

### 2.1. Theory of Planned Behaviour (TPB)

The TPB model offers a structured framework for predicting and explaining human behaviour by understanding beliefs and attitudes. This theory is a belief-based social-cognitive consumer theory that was developed as an extension to the theory of reasoned action [16]. The model suggests that people’s expectations and values about engaging in a behaviour form their behavioural, normative, and control beliefs. These beliefs, in turn, influence consumers’ attitudes, subjective norms, and perceived behavioural controls towards their purchase intentions and, ultimately, their purchasing behaviours.

The TPB has been applied extensively to food studies but rarely focuses on the intention to purchase or the consumption of ready meals. Among the few studies available, Olsen and Sijtsema [12] investigated the usefulness of integrating moral attitude into the TPB model when predicting intention to consume RTE meals in three countries: Norway, the Netherlands, and Finland. They found that the intention to consume RTE meals was partly driven by a moral attitude. According to their study, in all three countries, the feeling of moral obligation, operationalised as a negative feeling of guilt, had a negative effect on the intention to consume RTE meals. Another study by Mahon and Cowan [6] included a measure of habit into their TPB study model to investigate the consumption of ready meals and the purchase of takeaways by British consumers. Their findings revealed that including habit in the regression improved the model’s predictive power by 26%, demonstrating that habit is the strongest predictor of the intention to consume ready meals.

### 2.2. Theory of Consumption Value (TCV)

Consumption value is the perceived value acquired from product consumption [17,18]. The theory of consumption value was developed by Sheth and Newman [17], who reported that the consumers’ choice behaviour is influenced by several consumption values that include functional value, emotional value, social value, epistemic value, and conditional value. Each of these five values influenced the consumers’ choices and purchase decisions in different ways.

The TCV has been applied in several food-related research contexts, and recent literature suggests that the consumption value leads to purchase intention [19,20,21,22] and other behavioural intentions [23,24]. Al-Waseti and ve İrfanoğlu [20] used the consumption value theory to determine the consumer purchase intention towards organic food. They found significant relationships between various factors and the intention to purchase organic food. For instance, functional value (quality + price) and emotional value were found to have a positive direct impact, whereas social value had a negative impact on the purchase intention of organic food.

### 2.3. Linking TPB and TCV Constructs

The integration of the TPB and TCV offers a comprehensive approach to understanding consumer behaviour and purchase decisions, especially in the context of purchasing meat-based chilled ready meals. The TPB emphasises the formation of intention, while TCV highlights the significance of consumption values in predicting purchase behaviour. Several researchers point out the limitations of both TPB and TCV. For instance, TPB overlooks the role of emotions in shaping intentions and decision-making, as well as the importance of human needs prior to taking action [25]. Meanwhile, TCV is a narrower framework that primarily focuses on how values influence choice behaviour and identifies perceived values related to product purchases, but it fails to explain the underlying causes of those values [26]. To address these research gaps and achieve a more comprehensive understanding of the complexities of consumption behaviour, we have integrated TPB and TCV to examine purchase intentions for ready meals. Previous research has also recognised the complementary aspects of these theories [26,27,28]. In this study, we anticipate that our integration of TCV into the TPB model will be both relevant and appropriate. This integrated research framework will provide comprehensive and complementary insights on consumers’ purchase intentions for meat-based chilled ready meals in New Zealand.

### 2.4. Hypotheses Development and Proposed Research Model

**Figure 1:**
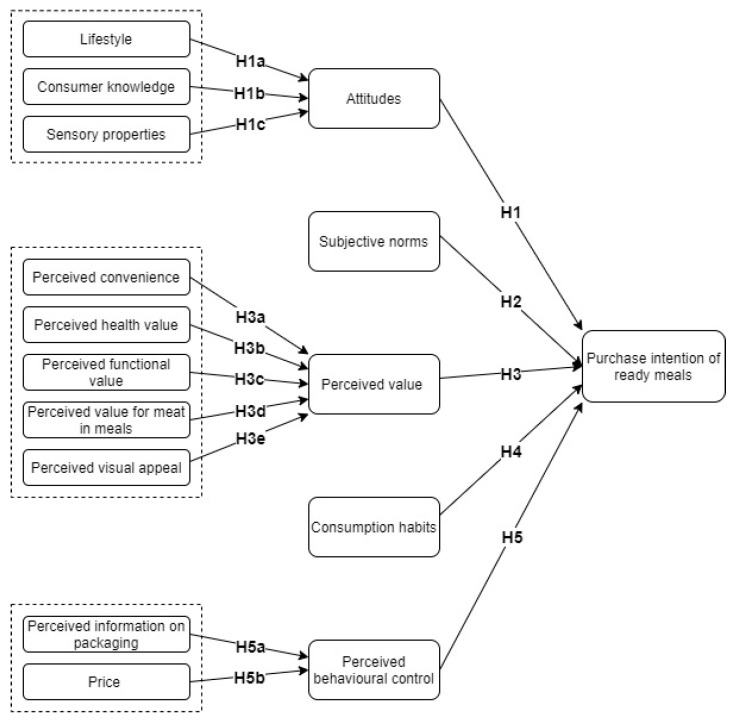
Conceptual model developed for this study.

In this study, the TPB model was extended to incorporate consumer choice behaviour and consumption habits. The proposed model and hypotheses for the study are presented in Figure 1. The model proposes possible links between five latent variables, namely attitudes, subjective norms, consumer choice behaviour, consumption habits, and perceived behavioural control. Additionally, consumer demographics are taken into consideration because buying decisions can be influenced by personal factors such as age or stage of life, family size, education, occupation, cultural background, and economic situation.

This section provides a detailed explanation and supporting rationale for the hypotheses proposed in this study. Figure 1 shows the conceptual model that was developed to depict the relationships between the variables.

Attitude can be defined as either a positive or negative evaluation of the performance of a particular behaviour [29] based on the consequences of engaging in a particular behaviour and the corresponding favourable or unfavourable judgment about the possible consequences [30]. According to Kotler and Armstrong [31], lifestyle influences consumers’ attitudes, buying behaviours, and decisions. Busy lifestyles and the associated time pressure or stress may lead consumers to prioritise convenience in their purchases [32]. This can translate into a preference for online purchase, subscription services, or in-store shopping of ready meals that save time and effort.

**H1:**  *Consumer attitude has a significant relationship with the consumer willingness to purchase meat-based chilled ready meals*.

Consumers lack knowledge about the nutritional or functional value of ready meals [1,33]. Most ready meals are considered unhealthy by consumers, although the ready meals available in the market now are healthier and more varied [34].

The sensory properties of chilled ready meals play a crucial role in shaping consumer attitudes and preferences. Consumer attitudes and beliefs and/or previous experiences contribute to their expectations [35]. When evaluating a particular chilled ready meal, the association with the homemade version aids consumers in envisioning the product [36]. Therefore, when consuming the meal, the actual sensory characteristics of the chilled ready meal should also match their expectations. At present, limited information is available regarding the influence of sensory properties on consumer attitudes, specifically in the context of purchasing meat-based chilled ready meals.

Therefore, we predict that consumers’ attitudes toward meat-based chilled ready meals will be influenced by lifestyle, consumer knowledge, and sensory properties. Hence,

**H1a:**  *Lifestyle positively and directly influences the attitude toward meat-based chilled ready meals*.

**H1b:**  *Consumer knowledge of meat-based chilled ready meals positively and directly influences attitudes*.

**H1c:**  *Sensory properties of meat-based chilled ready meals positively and directly influence attitudes*.

The subjective norm refers to the perceived social pressure to approve and adopt a certain behaviour [30], which means that if the purchase of ready meals becomes more socially accepted and associated with higher social status, it is likely to result in an increased demand for ready meals on the market. This can be understood within the framework of the consumption value theory, specifically in social value which reflects the benefits a consumer perceives in terms of social approval, social status, or group affiliation. It includes the perception of how a product influences a consumer’s social standing or acceptance among peers [17].

**H2:**  *Subjective norms have a significant relationship with the consumer willingness to purchase meat-based chilled ready meals*.

The theory of consumption values (TCV) is the leading framework for understanding how consumers perceive value [37]. According to Sheth and Newman [17], consumer choice decision making is influenced by multiple consumption values. These values make differential contributions in any given choice situation and are independent. Sheth identified five key value dimensions that significantly impact consumer preferences: conditional value, functional value, social value, emotional value, and epistemic value. The consumption values associated with a product play a critical role in shaping consumers’ behavioural intentions [38]. Therefore, we consider consumption values to be direct predictors of the consumer choice behaviour of meat-based chilled ready meals.

**H3:**  *Consumer choice behaviour of chilled ready meals has a significant relationship with the consumer willingness to purchase meat-based chilled ready meals*.

For our study, we exclude social value, as it aligns with the subjective norm in the TPB, which already captures social influences, including societal expectations and normative beliefs. The subjective norm considers the influence of societal expectations, social approval, and perceived pressure, which overlap with the concept of social value. Introducing social value as a separate construct could lead to redundancy, as both subjective norm and social value would essentially capture similar underlying social influences on behaviour.

Conditional value refers to the perceived worth of a product based on specific contexts or circumstances [17]. Chilled ready meals are often selected for their convenience, allowing consumers to save time and effort in meal preparation. Consequently, when evaluating these meals, consumers’ purchase intentions are shaped by the conditional value associated with their ability to meet immediate convenience needs.

Functional value relates to the practical benefits that a product offers, influencing decision-making and purchase intentions [38]. Chilled ready meals can be customized to address various dietary needs, providing functional benefits such as weight loss, muscle gain, and quick nutrient absorption. These attributes make them particularly appealing to specific consumer segments, including fitness enthusiasts, athletes, and elderly individuals.

Evaluating the value of meat separately from its functional value is crucial for understanding consumers’ choice behaviour towards meat-based chilled ready meals. Meat often serves as a focal point in purchase decisions, influenced by factors like quality, source, and health perception [39]. Its unique attributes, such as nutritional benefits and alignment with dietary trends, merit independent examination. This approach helps researchers understand the value of meat in meals, offering deeper insights into how it enhances the consumer choice behaviour of meat-based chilled ready meals and influence purchasing decisions.

Epistemic value is associated with the desire for knowledge and new experiences [17]. As consumers become more health-conscious, they actively seek food options that align with the latest dietary trends and innovations. Chilled ready meals that promote well-being, such as those low in calories, sodium, and fat but high in protein or essential nutrients, can spark consumer curiosity. This epistemic value motivates consumers to explore healthful options that enhance their well-being, thus increasing their intention to purchase. Additionally, knowledge about the health benefits of meat, including its essential amino acids, can further elevate consumers’ perception of its value in chilled ready meals.

Emotional value encompasses the feelings and psychological benefits derived from a product, which influence overall satisfaction and purchase intentions [17]. The visual appeal of a meal significantly contributes to its emotional value. Well-presented chilled ready meals can evoke feelings of anticipation and satisfaction even before consumption. Attractive packaging and vibrant ingredients can generate excitement and desire, making consumers more inclined to choose those products.

In the context of this study, it is predicted that the consumers’ choice behaviour of meat-based chilled ready meals will be influenced by conditional value, epistemic value, functional value, the perceived value of meat in the meal, and emotional value.

**H3a:**  *Conditional value directly influences the consumer choice behaviour towards meat-based chilled ready meals*.

**H3b:**  *Epistemic value directly influences the consumer choice behaviour towards meat-based chilled ready meals*.

**H3c:**  *Functional value directly influences the consumer choice behaviour towards meat-based chilled ready meals*.

**H3d:**  *Perceived value of meat in the meal directly influences the consumer choice behaviour towards meat-based chilled ready meals*.

**H3e:**  *Emotional value directly influences the consumer choice behaviour towards meat-based chilled ready meals*.

Habitual decisions are usually made routinely with little or no conscious effort. This is the opposite of extended decision-making where information is sought and thought through. Habitual behaviour poses a challenge for marketers aiming to disrupt established consumer routines when introducing new products [40]. Moreover, Mahon and Cowan [6] show that the predictive power of the TPB model they used in their study about the consumption of ready meals and purchase of takeaways by British consumers, was improved by the addition of the measure of consumer habit in their hypothesis testing.

**H4:**  *Consumption habits have a significant relationship with consumer purchase intention of meat-based chilled ready meals*.

Behaviour is affected by adequate resources and the ability to control any barriers to behaviours [41]. According to a previous research, barriers such as price, availability, trust, and information play crucial roles in limiting consumers’ ability to make the consumption choices they desire [42]. These factors are directly linked to the consumer’s perceived behavioural control (PBC), which influences their intention to purchase.

Price has been shown to impact purchasing decisions due to its direct influence on affordability and accessibility [43,44]. Similarly, packaging information, including product details and labelling, has been found to enhance consumers’ perceived control by offering clarity, trustworthiness, and ease of decision-making, particularly in food-related purchases [45,46,47]. In this context, increased access to resources (e.g., money and information about ready meals) and fewer barriers (such as lower prices) are likely to improve consumers’ perceived behavioural control, thereby strengthening their purchase intentions. Specifically, we hypothesize that the perceived behavioural control over the intention to purchase meat-based chilled ready meals will be significantly influenced by factors such as packaging information and price. A previous research has shown that similar factors, such as labelling and price, have a significant impact on consumers’ intention to purchase organic food products through their influence on PBC [42].

**H5:**  *Perceived behavioural control has a significant relationship with consumers’ intentions to purchase meat-based chilled ready meals*.

**H5a:**  *Perceived information on the package of the meat-based chilled ready meals directly influences the perceived behavioural control on ready meals*.

**H5b:**  *Price of the meat-based chilled ready meals directly influences consumers’ perceived behavioural control over their purchase decisions*.

## 3. Materials and Methods

### 3.1. Focus Group Interviews

To explore consumer perceptions of meat-based chilled ready meals, qualitative data were gathered through focus group interviews. Two focus groups were conducted, each consisting of 4–6 frequent consumers of meat-based chilled ready meals. Participants were selected based on their regular consumption of these products and diverse demographic backgrounds, including age and gender. The focus groups were moderated, and the discussions were structured around key questions focused on participants’ perceptions of the sensory qualities of meat-based chilled ready meals, such as taste, aroma, texture, appearance, freshness, and overall satisfaction. Participants were encouraged to express the importance of these sensory factors in comparison to other considerations, including price, nutrition, and convenience. The discussions were open-ended, allowing for a deep exploration of individual preferences and perceptions towards meat-based chilled ready meals.

The data were analysed using thematic analysis, which identified key recurring themes, including a notable dissatisfaction with the sensory quality of ready meals, particularly in comparison to freshly cooked meals. These insights were then used to inform the development of the survey instrument, ensuring that the relevant themes were reflected in the survey questions.

### 3.2. Development of the Survey Instrument

The online questionnaire was prepared using Qualtrics (Qualtrics, Provo, UT, USA) and included questions related to consumer demographics (gender, age, annual household income, educational qualifications, working status, occupation, annual household income, ethnicity, type of household, and location of residence in New Zealand), and six constructs (attitude, subjective norms, perceived behavioural control, intention to purchase, measures of consumer choice behaviour, and consumption habits of meat-based chilled ready meals). Each section of the survey was clearly labelled with headings corresponding to the relevant construct, helping respondents understood the focus of each section. To maintain focus on the meat-based chilled ready meal category, respondents were reminded at the beginning of each section to consider meat-based chilled ready meals when answering the questions. All the survey measurement items were adapted from previous relevant studies and validated scales as shown in Appendix A.1. The survey items were measured on a seven-point Likert scale ranging from strongly disagree (1) to strongly agree (7). A pilot survey was conducted with 24 participants to check the understanding and validity of the question statements before data collection. Some wording was refined, and some statements were rephrased to improve clarity.

### 3.3. Sampling and Survey Distribution

The link of the online questionnaire was emailed or shared with participants via social and professional networks. Data were gathered in New Zealand through a convenience sampling technique. All the participants were New Zealand residents. Since the survey was administered via the internet and social media, there was a limitation for individuals who did not have internet access or chose not to respond to social media. Even though we lacked control over the sample, it was the least expensive and least time-consuming option compared to other sampling techniques. Belonging to the target population ourselves, we attempted to leverage our network and asked specific respondents to encourage other individuals to participate in the survey. This is known as snowball sampling [48,49]. Incomplete responses were dropped from the analysis.

Ethics approval for this study was granted by the Auckland University of Technology Ethics Committee (AUTEC Reference number 20/285). Participants were required to be consumers of meat-based chilled ready meals, 18 years or older, and voluntarily agreed to participate after reviewing the participant information sheet. Those who did not meet these criteria were excluded from the study.

The questionnaire was completed by 543 individuals, although only 85% of the questionnaires were fully completed for all demographics and TPB questions. A priori power analysis (G*Power 3.1) was conducted before the survey using the expected power (1-β error probability) of 0.80 as suggested by Cohen [50] and Hair and Risher [51], an alpha (α) error probability of 0.05, sixteen predictors, and an effect size of 0.15 to represent the medium effect size (R^2^ of 0.13). The minimum required sample size was 142, so the final sample of 464 was large enough to detect medium effects. The socio-demographic profile of the survey respondents is summarised in Table 1.

### 3.4. Statistical Analysis

In this study, the conceptual research model was analysed with XLSTAT 2020.5.1. using partial least squares path modelling (PLSPM). The PLS model used in the analysis is a component-based structural equation modelling approach. This was preferred over the traditional covariance-based approach because PLS can handle small sample sizes as long as there is adequate statistical power, and any outliers or missing data have been identified and treated [52]. Only completed survey responses with no missing data were used in the data analysis. This analysis captured both the relationship between the constructs and their indicators, as well as the relationship between latent variables. PLS can also handle complex models. A previous study that used PLS had an average of 8 latent constructs, 27 indicators, and 11 structural relationships per model [52]. The current study used a model that had 16 latent constructs, 59 indicators and 15 structural relationships, suggesting that the model is complex enough. Therefore, PLS was appropriate in analysing the model. T-tests were conducted to evaluate the significance of the path coefficients in the model, using a significance level of 0.05.

## 4. Results and Discussion

### 4.1. Measurement Model Analysis: Reliability and Validity

The measurement model specifies the relationship between a construct and its observed indicators and provides quantitative measures of the constructs’ validity and reliability [53]. It is expected that the observed indicators measure the same underlying latent variable. These observed variables must be unidimensional where they practically belong to one construct indicating the same latent variable [54]. The unidimensionality of the model constructs is shown in Appendix A.2. The eigenvalue of the second vector has consistently been found to be below 1 for all constructs, indicating a unidimensional structure for every construct.

Table 2 shows the reliability and validity measurements, including Cronbach’s alpha coefficients, Dillon-Goldstein’s rho (D.G. rho), and average variance extracted (AVE) scores for all constructs. The internal consistency of the model is acceptable because the Cronbach’s alpha ranged from 0.702 to 0.934, exceeding the threshold of 0.70 [55] for most variables except for three control variables; price (0.501), epistemic value (0.445), and perceived behavioural control (0.645). However, the D.G. rho reliabilities for all the constructs ranged from 0.728 to 0.958, exceeding the recommended 0.70 minimum [56]. The convergent validity of each construct was further tested using the average variance extracted (AVE), which describes the amount of variance that can be explained by items compared with the variance caused by measurement error. Constructs that have AVE values greater than 0.5 are said to have convergent validity or unidimensionality [57,58]. In the measurement model shown in Table 2, the AVE values for 13 out of 16 constructs were above the required minimum level of 0.50, except for perceived behavioural control (0.373), epistemic value (0.474), and price (0.497). To assess multicollinearity, we conducted a thorough examination of the correlations between the constructs in the model. The correlation matrix did not reveal any significant multicollinearity issues that would affect the validity of the model.

Although the Cronbach’s alpha and AVE were low for three of the constructs, they were not dropped out of the model since they had an acceptable D.G. rho of more than 0.70 (Table 2), an acceptable overall model fit (Table 3), and all the standardised factor loadings in the model were statistically significant (Table A2) and higher than 0.50 [59]. This indicates satisfactory composite reliability, so they were retained in the model.

### 4.2. Structural Model Analysis: Goodness of Fit

The conceptual research model of this study had a strong explanatory value for the intention to purchase meat-based chilled ready meals. The model fit indices assessed how well the model explains the data. The relative goodness of fit (GoF) for the overall model (Table 3) was 0.87, which is above the acceptable threshold of 0.80. Hence, the proposed model explains a significant number of relationships in the data.

The R^2^ value represents the predictive power of the model. The model was successful in predicting the intention to purchase meat-based chilled ready meals. This model explained 62% of the variation in the data. The unstandardised (original) R^2^ values for attitudes, consumer choice behaviour, and perceived behavioural control were 0.18, 0.30, and 0.14, respectively. These values indicate the proportion of variance in the dependent variable explained by the independent variables. However, to have a more accurate understanding of how well the model fits the data, the standardised R^2^ values through bootstrapping were calculated. The standardised R^2^ values for attitudes, consumer choice behaviour, and perceived behavioural control were 0.20, 0.32, and 0.17, respectively. These standardised values provide a better indication of the effect size and the strength of the relationship between the independent and dependent variables. It is important to note that R^2^ values higher than 0.10 are typically considered adequate for a latent construct [60,61]. In the current study, all three constructs (attitudes, consumer choice behaviour, and perceived behavioural control) exceeded this threshold. This suggests the model satisfactorily explains each construct. Furthermore, values between 0.33 and 0.67 indicate a moderated effect [61,62]. While the standardised R^2^ values in this study did not fall within this range, it is valuable to assess the effect size and potential moderating factors when interpreting the results.

### 4.3. Path Analysis and Hypotheses Testing

Table 4 summarises the findings of the structural model. Path coefficients are useful for understanding the magnitude and direction of the relationships among variables in the model [63]. They inform the amount of variance in the outcome variable that can be explained by each predictor variable and represent the magnitude of the effect of a change in one variable on another.

Bootstrap validation of path coefficients was carried out. This involved resampling the data 100 times with a sample size of 464 and running the analysis on each resampled dataset. This process allowed us to assess the stability and significance of the path coefficients across multiple iterations. The validation results indicate the likelihood of observing the original data in comparison to the bootstrapped samples. According to the bootstrap validation, all paths in the model were found to be valid and fit for interpretation.

The T-test results, detailed in Table 4, support all the main hypotheses (H1, H2, H3, H4, and H5) and their associated sub-hypotheses. However, two sub-hypotheses (H3c and H5a) were not supported. Specifically, this lack of support indicates that the functional value and the information provided on the packaging did not have a significant direct effect on consumer choice behaviour and perceived behavioural control.

#### 4.3.1. Attitudes

To assess the impact of lifestyle on attitudes, the study included question statements such as “I purchase ready meals for consumption when I finish work late” and “Ready meals are less stressful than preparing a cooked meal from scratch and help me lead a relaxed lifestyle”. These statements highlight that perceptions of a busy lifestyle and the desire for convenience contribute to more positive attitudes towards meat-based chilled ready meals. The findings revealed that consumers’ attitudes towards these meals were positively influenced by their lifestyle (H1a; β = 0.19, *p* < 0.001). This aligns with findings from Kotler and Armstrong [31], which also identified lifestyle as influencing buying behaviour and decisions.

This study revealed that consumers’ attitudes towards meat-based chilled ready meals were positively influenced by their knowledge about these products (H1b; β = 0.31, *p* < 0.001). However, many consumers often lack awareness about the nutritional and functional benefits of ready meals [1,33]. Despite improvements in the healthiness and versatility of ready meals, our survey indicated that many consumers still perceive them as unhealthy and poorly balanced nutritionally (mean scores for each question statements are detailed in Appendix A.2).

During initial focus group interviews with ready meal consumers in this study, a recurring theme was their dissatisfaction with the sensory quality of ready meals. Many participants considered ready meals as inferior to freshly cooked meals. Consumers rated the question statements indicating how the meals’ taste, aroma, texture and appearance, as well as fresh cooked quality, as very highly important to them. Together, these sensory properties positively influenced (H1c; β = 0.11, *p* < 0.05) their attitudes towards meat-based ready meals. This finding is consistent with Reed and McIlveen-Farley [64] who found that the taste of chilled ready meals was a more important factor than price when it came to consumer preferences. This highlights that the sensory appeal of products should not be underestimated. These insights from our research, and from previous studies, emphasise the need for the future development of ready meals to focus on sensory factors. Creating ready meals with a desired sensory quality that closely resembles freshly cooked meals can have a positive impact on consumers’ overall perception and attitudes towards chilled ready meal purchase intention.

Although lifestyle, consumer knowledge, and sensory properties positively influenced attitudes, overall attitudes had a negative effect on purchase intention of meat-based chilled ready meals (H1; β = −0.08, *p* < 0.05). This finding suggests that simply promoting a positive attitude towards meat-based chilled ready meals may not be sufficient to drive purchase behaviour. Marketers may need to focus on addressing specific barriers that hinder purchase decisions, even among consumers with positive attitudes. These barriers could include concerns related to freshness, healthiness, and alignment with activities or lifestyle preferences. To overcome these challenges, product reformulation, better alignment with current consumer trends and innovative packaging design and solutions could be implemented. These efforts could help mitigate the negative impact of attitudes on purchase intentions and make meat-based chilled ready meals more appealing to consumers.

#### 4.3.2. Subjective Norms

Subjective norms, as hypothesised in H2, had a direct significant effect on consumers’ intentions to purchase meat-based chilled ready meals. The path coefficient for this relationship was calculated as 0.29 (H2; β = 0.29, *p* < 0.001), indicating a significant influence. This means that participants’ intentions to purchase ready meals can be predicted by their perceptions of social influence, specifically the opinions and attitudes of friends, family and people who are important to them. The key social factors include reference groups, family, and role and status [65]. According to Kotler and Armstrong [31] consumers are part of various social, membership, or reference groups. Reference groups, such as family, close friends, neighbours, colleagues, or other associations, play a crucial role in shaping consumers’ self-image and behaviours. These groups significantly impact consumers’ purchasing decisions, including those related to ready meals [31].

It is also important to consider the significant role that personal factors can play. Several studies suggest that the influence of personal factors, such as attitudes and product attributes, can often outweigh the impact of social norms or pressures in shaping consumer behavioural intentions [65,66]. Hence, both social influences and individuals’ own beliefs, habits, and perceptions of product-related attributes can influence consumers’ intentions to purchase ready meals.

#### 4.3.3. Consumer Choice Behaviour of Meat-Based Chilled Ready Meals

According to the theoretical framework of consumer choice behaviour developed by Sheth, Newman [17], consumer choice is regarded as a function of multiple ‘consumption value’ dimensions that ultimately influence purchase intentions and decisions. In the marketing context, efforts are made to influence and increase the perceived value of a product or service through qualities such as aesthetic design, accessibility, or convenience. Consumer choice behaviour had a significant influence on consumers’ intentions to purchase meat-based chilled ready meals, supporting hypothesis H3 (β = 0.10, *p* < 0.01). As expected, conditional value (H3a; β = 0.29, *p* < 0.001) was the most positive influential factor in determining the consumer choice behaviour of meat-based chilled ready meals. It contributed to 40% of the overall R^2^ value. This could be attributed to the benefits of convenient meals in saving time, physical and mental energy. Several studies have outlined the importance of convenience in meal selection, purchasing, and preparation [67,68].

The second important element influencing the consumer choice behaviour of meat-based chilled ready meals was the perceived value of meat itself (H3d). This element contributed to 23% of the R^2^ of consumer choice (β = 0.19, *p* < 0.001). Interestingly, despite the recent trend towards vegetarian diets [69], consumers perceived better value in ready meals containing meat.

Although consumers have different beliefs about the nutritional or health value of ready meals, it was found that epistemic value (H3b) positively affected the intention to purchase meat-based chilled ready meals. This indicates that consumers value the epistemic values associated with these products (β = 0.11, *p* < 0.05). The epistemic value of foods is a hidden characteristic, which cannot be directly perceived through our senses. However, it is becoming increasingly valued by consumers [70]. According to Van der Horst and Brunner [1], some consumers perceived ready meals as containing more vitamins and nutrients, leading to more positive beliefs about the health value of ready meals and, ultimately, influencing their ready meal intake [1].

In addition to epistemic value and the value of meat in the meal, the emotional value of ready meals also plays a role in influencing consumer intentions and their perception of value. Emotional value (H3e) was found to be a positive predictive factor of consumer choice behaviour (β = 0.18, *p* < 0.001). This becomes crucial when consumers cannot directly sense the flavour or texture of the product because it is pre-packaged. This includes elements such as package design, graphics, and the visual attributes of the ready meal, including its appearance of being freshly cooked. In this study, 85.6% of respondents agreed (rating score > 5) with the statement “Ready meals that have a “freshly cooked” appearance would positively influence my purchasing intention”. This attests to the positive influence observed, indicating that ready meals characterised by freshness, visual appeal and a ‘freshly cooked’ aesthetic exerted a positive effect on consumers’ intentions to purchase.

The functional value (H3) had no impact on the consumer choice behaviour of meat-based chilled ready meals (β = 0.09, *p* > 0.05). Basaran and Aksoy [71] also reported that functional value did not affect the willingness to pay more, but it had a greater impact on repeat purchase behaviour. In our study, we specifically sought to examine whether consumers viewed meat-based chilled ready meals as offering functional benefits, such as weight loss, muscle gain, or quick nutrient absorption. These attributes could be particularly appealing to specific consumer segments, such as fitness enthusiasts, athletes, or older adults. However, the unsupported hypothesis (H3c) suggests that functional value did not play a direct role in consumer choice behaviour. This indicates that in the context of meat-based chilled ready meals, consumers may not view functional value as a key differentiator.

#### 4.3.4. Consumption Habits

Consumer habit had the strongest impact on the intention to purchase meat-based chilled ready meals (H4; β = 0.54, *p* < 0.001). This result suggests that habit is the most important predictor of consumers’ intentions to purchase ready meals. To measure the influence of consumption habits on the intention to purchase ready meals, four question statements were used, including “I regularly purchase ready meals during my weekly shopping”; “I usually consume ready meals as my main meal”; “A large proportion of my weekly food consumption is ready meals”; and “I eat ready meals at least once a week”. It is important to note that not all subjects responded with higher rating scores (>5) for consumption habit question statements. Rather, those who had higher ratings (31.5%) also expressed their willingness or intention to purchase a ready meal in the next four weeks. On the other hand, those who responded with lower rating scores (<4) on those statements also had lower ratings of the intention to eat a ready meal in the following four weeks. This suggests a strong relationship between consumption habits and purchase intentions.

Several studies have shown that the habit can influence the intention to purchase or consume ready meals [1,6,72,73]. For example, Mahon and Cowan [6] found that including a measure of habit in their regression model increased the predictive power, indicating the importance of habit as the most important predictor of intention to consume ready meals. Similarly, in our model, strong path coefficients were observed between consumer habits and purchase intention, further supporting the influence of habit on consumers’ purchase intentions.

#### 4.3.5. Perceived Behavioural Control

Perceived behavioural control (H5) had a significant influence on the intention to purchase meat-based chilled ready meals, with a path coefficient of 0.13 (*p* < 0.001). When examining the factors that influence perceived behavioural control, it was found that the price had a significant positive influence (H5b; β = 0.36, *p* < 0.001), while the perceived information on the packaging had no impact (H5a; β = 0.09, *p* > 0.05). The impact of ‘Price’ on perceived behavioural control was gauged through three statements: “Price of the ready meal is important to me”, “I am willing to pay a premium price if the ready meals meet my nutritional needs”, and “I am happy to pay a premium price if the ready meal is of excellent quality”. These statements were designed to capture both the significance of price and the consumers’ willingness to pay a premium for meat-based chilled ready meals that align with their quality and nutritional requirements. The strong path coefficient (β = 0.36) in this study indicates that price was the most influential factor in predicting perceived behavioural control. Moreover, the price accounted for 90% of the variation the perceived behavioural control construct.

The finding that perceived information on packaging had no significant effect on perceived behavioural control does not diminish the value of nutritional information on packaging. Consumers often perceive this information as a credible source when selecting food products [74]. However, in the context of meat-based chilled ready meals, it appears that other factors, such as price, have a stronger influence on consumers’ perception of control over their behaviour. This aligns with previous findings discussed by Weatherell and Tregear [75], wherein the priorities of consumers in food selection emphasised the greater importance of price and convenience in comparison to image-related factors, such as packaging and brand. One possible explanation for this finding is that New Zealand consumers may place more trust in alternative sources of information, such as online reviews, recommendations, or word-of-mouth, rather than relying solely on packaging. As the food industry evolves, digital platforms have become an increasingly important source of information, guiding consumers’ purchasing decisions. Additionally, frequent buyers of meat-based chilled ready meals may not feel the need to read packaging information, particularly when they are purchasing in a rush, or simply looking to satisfy hunger or a meal need. These behaviours reflect the typical characteristics of ready meal consumers, who often prioritize convenience and efficiency over detailed product information.

## 5. Conclusions

This study employs a novel integration of the consumption value theory and theory of planned behaviour to examine the factors influencing the consumers’ intention to purchase meat-based chilled ready meals in New Zealand. The findings highlight the consumers’ responses towards their intention to purchase these meals, as evidenced by consistent and significant influences identified through path coefficients and hypothesis testing. Key factors such as lifestyle, consumer knowledge, sensory properties, subjective norms, consumer choice behaviour, consumption habits, and perceived behavioural control were identified as crucial determinants in the decision-making process related to these products. These results provide valuable insights into the complex nature of consumer decision-making, particularly in the context of meat-based chilled ready meals.

By examining the interplay between various psychological, social, and emotional factors, this study extends the current theoretical understanding of consumer behaviour in the context of chilled ready meals. The findings support the idea that consumer decisions are not solely based on product attributes, but are also influenced by broader lifestyle choices, knowledge, and social norms. This study therefore contributes to the literature by broadening the scope of both theories, suggesting that they are highly applicable for studying food-related consumer behaviours.

From a practical standpoint, the study provides important recommendations for marketers, manufacturers, and researchers in the chilled ready meal industry. The emphasis on consumer education, particularly regarding ingredients, packaging, and product quality, highlights the need to address knowledge gaps in order to positively influence purchase intentions. Additionally, the study identifies sensory appeal as a crucial factor in shaping attitudes towards meat-based chilled ready meals, suggesting that product developers should focus on improving the sensory quality (taste, texture, aroma) of these meals. By understanding the underlying factors that influence consumer behaviour on chilled ready meals, businesses can unlock avenues for innovation and growth, ensuring their sustained presence in the market.

While this study provides valuable insights, several limitations must be acknowledged. Firstly, this study was conducted exclusively in New Zealand, limiting the generalizability of the findings to other regions or cultural contexts. Secondly, the survey was administered via the internet and social media, which may have excluded certain segments of ready meal consumers, particularly those without internet access or those who do not engage with social media. Future research could explore alternative data collection methods, such as in-person surveys or phone interviews, to reach a broader and more diverse audience. Finally, this study does not account for all potential factors influencing consumer preferences and purchase intentions, such as demographic variables or broader socio-economic trends. Further research could expand the scope to include additional variables and explore their influence on consumer behaviour.

## Figures and Tables

**Table 1:** Sociodemographic profile of respondents.

Variable\Statistic	Categories	Frequencies	Percentage (%)
Gender	Female	321	69.2
	Male	143	30.8
Age range	Below 20	9	1.9
	20–29	107	23.1
	30–39	118	25.4
	40–49	92	19.8
	50–59	75	16.2
	60 or more	63	13.5
Highest level of education	Secondary school qualification—Not completed	13	2.8
	Secondary school qualification—Completed	55	11.9
	Certificate/Diploma	54	11.6
	Bachelors’ degree	135	29.1
	Postgraduate cert/diploma	8	1.7
	Masters’ degree	104	22.4
	PhD	95	20.5
Working status	Casual	15	3.2
	Full time	286	61.6
	Part-time	83	17.9
	Retired	12	2.6
	Self-employed	17	3.6
	Studying	44	9.5
	Unemployed	7	1.5
Occupation	Accommodation and Food Services	22	4.7
	Administrative and Support Services	34	7.3
	Agriculture, Forestry and Fishing	49	10.6
	Art, Sport and Recreation	7	1.5
	Construction	4	0.9
	Education and Training	107	23.1
	Electricity, Gas, Water and Waste services	1	0.2
	Financial and Insurance Services	5	1.1
	Health Care and Social Assistance	14	3.0
	Housewife/Househusband	8	1.7
	Information, Media, and Telecommunication	12	2.6
	Manufacturing	18	3.9
	Professional, Scientific, and Technical Services	150	32.3
	Public administration and Safety	6	1.3
	Transport, Postal, and Warehousing	2	0.4
	Wholesale and Retail Trade	21	4.5
	Other Services	4	0.9
Annual income before tax (NZD) ^†^	Below 10,000	52	11.2
	10,001–30,000	72	15.6
	30,001–50,000	60	12.9
	50,001–70,000	99	21.3
	70,001–100,000	115	24.8
	Above 100,001	66	14.2
Type of household	Single person residing alone	62	13.4
	Shared house with siblings	2	0.4
	Shared house with unrelated individuals	77	16.6
	Family home-one parent with child(ren)	11	2.4
	Family home-couple with no children	134	28.9
	Family home-couple with child(ren)	150	32.3
	Extended family/multi-generation home	28	6.0
Ethnic group	European (including NZ European)	318	68.5
	Māori	18	3.9
	Pacific	8	1.7
	Asian	99	21.3
	Middle Eastern/Latin American/African	17	3.7
	American	4	0.9

^†^ All annual income figures are in New Zealand Dollars (NZD). Conversion rates used as of 24 February 2025: 1 NZD = 0.57 USD and 1 NZD = 0.55 EUR.

**Table 2:** Reliability and validity of measurements model (XLSTAT PLSPM software).

Latent Variable	Dimensions	Cronbach’s Alpha	D.G. rho (PCA) ^†^	Condition Number	AVE ^‡^
Sensory properties	5	0.897	0.924	3.822	0.696
Lifestyle	2	0.702	0.870	1.832	0.768
Consumer knowledge	3	0.710	0.844	2.322	0.569
Attitudes	4	0.754	0.847	3.024	0.584
Subjective norms	3	0.801	0.884	3.543	0.724
Perceived information on package	4	0.709	0.827	2.255	0.528
Price	3	0.501	0.759	1.718	0.497
Perceived behavioural control	5	0.645	0.777	2.579	0.373
Consumption habits	4	0.885	0.927	3.962	0.744
Emotional value	4	0.804	0.872	4.240	0.649
Conditional value	4	0.778	0.864	2.759	0.596
Epistemic value	3	0.445	0.728	1.435	0.474
Functional value	4	0.713	0.824	2.187	0.537
Perceived value of meat in meals	4	0.781	0.860	2.479	0.604
Consumer choice behaviour	4	0.775	0.860	2.669	0.602
Purchase intention	3	0.934	0.958	6.587	0.885

^†^ Dillon-Goldstein’s rho (Principal Component Analysis). ^‡^ Average variance extracted.

**Table 3:** Goodness of fit (GoF) index and model measurement.

	GoF	GoF (Bootstrap)	Standard Error	Lower Bound (95%)	Upper Bound (95%)
Absolute	0.435	0.445	0.014	0.414	0.477
Relative	0.870	0.856	0.014	0.829	0.884
Outer model	0.987	0.984	0.004	0.972	0.991
Inner model	0.881	0.870	0.014	0.847	0.898

**Table 4:** Path coefficients and hypotheses testing results.

Hypotheses	Hypothesised Path	Path Coefficients	Path Coefficients (Bootstrap)	Pr > |t|	Testing Results
H1a	Lifestyle → Attitudes	0.19	0.19	0.000	Supported
H1b	Consumer knowledge → Attitudes	0.30	0.31	0.000	Supported
H1c	Sensory properties → Attitudes	0.10	0.11	0.028	Supported
H3a	Conditional value → Consumer choice behaviour	0.29	0.29	0.000	Supported
H3b	Epistemic value → Consumer choice behaviour	0.10	0.11	0.027	Supported
H3c	Functional value → Consumer choice behaviour	0.08	0.08	0.064	Not supported
H3d	Perceived value of meat in meals → Consumer choice behaviour	0.19	0.19	0.000	Supported
H3e	Emotional value → Consumer choice behaviour	0.16	0.18	0.000	Supported
H5a	Perceived info on package → Perceived Behavioural Control	0.07	0.09	0.149	Not supported
H5b	Price → Perceived Behavioural Control	0.35	0.36	0.000	Supported
H1	Attitudes → Purchase intention	−0.07	−0.08	0.028	Supported
H2	Subjective norms → Purchase intention	0.28	0.29	0.000	Supported
H3	Consumer choice behaviour → Purchase intention	0.10	0.10	0.009	Supported
H4	Consumption habits → Purchase intention	0.54	0.54	0.000	Supported
H5	Perceived behavioural control → Purchase intention	0.14	0.13	0.000	Supported

## Data Availability

The original contributions presented in this study are included in the article. Further inquiries can be directed to the corresponding author.

## Abbreviations

The following abbreviations are used in this manuscript:

TPB	theory of planned behaviour
TCV	theory of consumption values
PLSPM	partial least squares path modelling
AVE	average variance extracted

## Appendix A

### Appendix A.1

**Table A1:** Consumer purchasing behaviour question statements and sources of adaption of question elements.

Construct	Manifest Variables	Question Statements (Seven-Point Likert Scale)	Source of Adoption
Conditional value (CON)	CON1	I can save time in the shop when I purchase chilled ready meals.	[76,77]
	CON2	Chilled ready meals are a good back up to have in the home when I have little time to prepare meals.	[6]
	CON3	Chilled ready meals are fast to prepare at home.	[6]
	CON4	It is easier for me to purchase a meat-based chilled ready meal than cooking a meal from scratch.	[77,78]
Epistemic value (EPV)	EPV1	I would be more likely purchase a meat-based chilled ready meal if it’s labelled as less salt and less fat.	[79]
	EPV2	When I purchase a meat-based chilled ready meal, I always look at nutritional information.	
	EPV 3	I like meat-based chilled ready meals if those promote my wellbeing and healthy lifestyle.	[80,81]
Perceived value of meat in meals (PMTV)	PMTV1	I would be more likely to purchase a chilled ready meal if it contains meat and vegetables as it provides me with better nutrition.	[80]
	PMTV2	I would be more likely to purchase a meat-based chilled ready meal as it promotes my health and well-being.	[7]
	PMTV3	I would expect chilled ready meals containing meat to be healthier and more nutritious than those without meat.	
	PMTV3	I would pay more if the chilled ready meals contain meat.	
Attitudes (ATU)	ATU1	Meat-based chilled ready meals are well balanced meals	
	ATU2	Meat-based chilled ready meals can provide my daily nutritional requirements.	
	ATU3	I would be happy to purchase meat-based chilled ready meals that would give sustained energy.	[82]
	ATU4	I think chilled ready meals containing meat would boost my mood over those without meat.	[82]
Functional value (FUN)	FUN1	It is great if the meat-based chilled ready meal has its own functional benefits (for example, weight loss, muscle gain, digestibility, fast absorption of nutrients, etc.).	
	FUN2	I prefer if the meat-based chilled ready meals have higher protein content.	
	FUN3	I prefer if the meat-based chilled ready meals are easy to digest.	
	FUN4	I will prioritize the functional benefits over other attributes of the meal when I am purchasing a chilled ready meal contain meat.	
Consumer knowledge on ready meals (CNKW)	CKNW1	I feel that I have clear understanding and knowledge of meat-based chilled ready meals.	[80]
	CKNW2	Most meat-based chilled ready meals have an acceptable standard of quality.	[18]
	CKNW3	I am knowledgeable about meat-based chilled ready meals I eat and how they can meet my nutritional need.	[80]
Consumer choice behaviour (CCB)	CCB1	Meat-based chilled ready meals can be good value for money when reasonably priced.	[6]
	CCB2	Meat-based chilled ready meals have acceptable standard of quality for paid price.	[18]
	CCB3	Meat-based chilled ready meals can be an economical meal.	
	CCB3	When I buy meat-based chilled ready meals, I would ensure that I am getting my money’s worth	[83]
Subjective norms (SUBN)	SUBN1	I’m happy to tell people in my social circle that I purchase meat-based chilled ready meals.	
	SUBN2	If people in my social circle recommend that I purchase meat-based chilled ready meals, I would purchase meat-based chilled ready meals.	[12,53]
	SUBN3	If people whose opinion I value recommend that I purchase chilled ready meals containing meat, I would purchase chilled ready meals containing meat.	[78]
Emotional value (EMV)	EMV1	Meat-based chilled ready meals that look fresh and appealing would positively influence my purchasing intention.	[79]
	EMV2	Meat-based chilled ready meals that have a “freshly cooked” appearance would positively influence my purchasing intention.	
	EMV3	Ready meal package design and graphics would positively influence my purchasing intention.	[53]
	EMV4	I expect the meat-based chilled ready meal inside the packaging to look the same as displayed on the packaging	
Perceived information on package (PINF)	PINF1	I would like to know nutrition facts, shelf life and ingredients before purchasing meat-based chilled ready meals.	[79]
	PINF2	I’m more likely to purchase a meat-based chilled ready meal that has a clear image of the meal on the packaging.	
	PINF3	When I purchase, I look for graphics that indicate meals contain low fat and salt.	
	PINF4	When I purchase, I look for health star ratings on the package.	
Sensory appeal (SEAP)	SEAP1	The taste of the meat-based chilled ready meal is very important for me	[81]
	SEAP2	The texture of the meat-based chilled ready meal is very important for me	[81]
	SEAP3	The aroma of the meat-based chilled ready meal is very important for me	
	SEAP4	The appearance of the meat-based chilled ready meal is very important for me	[68]
	SEAP5	The fresh cooked quality of the meat-based chilled ready meal is very important for me.	
Consumption habits (CHAB)	CHAB1	I usually consume meat-based chilled ready meals as my main meal.	[6]
	CHAB2	I eat meat-based chilled ready meal at least once a week	[6]
	CHAB3	I regularly purchase meat-based chilled ready meals during my weekly shopping.	[84]
	CHAB4	A large proportion of my weekly food consumption is meat-based chilled ready meals.	[83]
Perceived behavioural control (PBCN)	PBCN1	I make most of the decisions around what myself and my household consume.	[53]
	PBCN2	I do most of the household food shopping.	
	PBCN3	I am in control of the number of meat-based chilled ready meals I consume.	[12,78,79]
	PBCN4	I think it’s easy for me to buy meat-based chilled ready meals.	[12,78,79]
	PBCN5	I believe that I have the money resources and the ability to buy meat-based chilled ready meals.	[53]
Price (PRIC)	PRIC1	Price of the meat-based chilled ready meal is important to me.	[79]
	PRIC2	I am willing to pay a premium price if the meat-based chilled ready meals meet my nutritional needs.	[85]
	PRIC3	I am happy to pay a premium price if the meat-based chilled ready meal is excellent quality.	
Lifestyle (LFST)	LFST1	I purchase meat-based chilled ready meals for consumption when I finish work late.	[14]
	LFST2	Meat-based chilled ready meals are less stressful than preparing cooked meal from scratch and help me lead a relaxed lifestyle.	[14,80,86]
Purchase intension (PINT)	PINT1	I am willing to purchase meat-based chilled ready meals within next 4 weeks.	[53,78]
	PINT2	I intend to purchase meat-based chilled ready meals within next 4 weeks.	[53,78]
	PINT3	I plan to purchase meat-based chilled ready meals within next 4 weeks.	[53,78]

### Appendix A.2

**Table A2:** Mean, standard deviation, loadings, and communalities of manifest variables (XLSTAT PLSPM software).

Latent Variable	Manifest Variables	Mean	S.D.	Standardized Loadings	Loadings	Communalities	Standardized Loadings (BOOTSTRAP)	Standard Error	Lower Bound (95%)	Upper Bound (95%)
Purchase intention	PINT1	4.155	1.895	0.901	0.058	0.812	0.902	0.010	0.882	0.923
	PINT2	3.517	1.859	0.976	0.061	0.953	0.976	0.003	0.966	0.982
	PINT3	3.310	1.888	0.944	0.060	0.891	0.944	0.007	0.924	0.958
Attitudes	ATTU1	4.709	1.426	0.809	0.058	0.655	0.809	0.028	0.745	0.859
	ATTU2	3.744	1.672	0.780	0.058	0.609	0.777	0.032	0.705	0.839
	ATTU3	3.862	1.294	0.684	0.054	0.468	0.680	0.043	0.592	0.766
	ATTU4	4.144	1.351	0.776	0.071	0.603	0.774	0.034	0.696	0.840
Subjective norms	SUBN1	4.446	1.551	0.743	0.054	0.552	0.743	0.037	0.649	0.813
	SUBN2	3.972	1.470	0.917	0.063	0.840	0.916	0.013	0.869	0.934
	SUBN3	4.313	1.481	0.883	0.062	0.780	0.881	0.021	0.827	0.912
Perceived behavioural control	PBCN1	5.642	1.385	0.549	0.047	0.302	0.547	0.098	0.330	0.738
	PBCN2	5.478	1.709	0.590	0.062	0.348	0.579	0.109	0.332	0.777
	PBCN3	6.183	1.086	0.503	0.033	0.253	0.496	0.082	0.289	0.633
	PBCN4	4.767	1.551	0.810	0.077	0.655	0.803	0.054	0.664	0.881
	PBCN5	5.401	1.377	0.553	0.047	0.305	0.551	0.068	0.418	0.714
Emotional value	EMV1	5.310	1.214	0.871	0.060	0.758	0.873	0.026	0.805	0.918
	EMV 2	5.397	1.206	0.849	0.058	0.720	0.851	0.029	0.770	0.908
	EMV 3	4.836	1.377	0.837	0.066	0.700	0.840	0.027	0.764	0.892
	EMV 4	5.356	1.442	0.645	0.053	0.416	0.633	0.063	0.480	0.751
Conditional value	CON1	4.675	1.547	0.729	0.055	0.531	0.725	0.041	0.636	0.804
	CON2	5.231	1.552	0.845	0.064	0.713	0.844	0.021	0.800	0.887
	CON3	5.707	1.143	0.677	0.038	0.459	0.675	0.045	0.562	0.754
	CON4	4.690	1.764	0.826	0.071	0.683	0.826	0.028	0.745	0.886
Epistemic value	EPV1	4.552	1.601	0.783	0.066	0.614	0.787	0.051	0.664	0.903
	EPV 2	4.606	1.791	0.662	0.062	0.439	0.648	0.084	0.450	0.830
	EPV 3	4.972	1.577	0.608	0.050	0.370	0.598	0.092	0.377	0.750
Functional value	FUN1	4.636	1.465	0.823	0.070	0.677	0.826	0.031	0.743	0.888
	FUN2	4.780	1.324	0.677	0.052	0.459	0.672	0.057	0.520	0.779
	FUN3	4.860	1.283	0.738	0.055	0.545	0.745	0.044	0.639	0.827
	FUN5	3.897	1.481	0.682	0.059	0.466	0.667	0.076	0.467	0.807
Consumption habits	CHAB1	2.269	1.634	0.763	0.049	0.582	0.765	0.029	0.704	0.822
	CHAB2	2.558	1.906	0.927	0.070	0.859	0.927	0.009	0.907	0.946
	CHAB3	2.537	1.826	0.935	0.067	0.875	0.935	0.008	0.914	0.950
	CHAB4	1.804	1.319	0.812	0.042	0.660	0.812	0.023	0.766	0.859
Sensory properties	SEAP1	6.252	0.935	0.756	0.050	0.572	0.733	0.103	0.401	0.854
	SEAP2	6.086	0.961	0.821	0.055	0.674	0.808	0.060	0.666	0.904
	SEAP3	5.940	1.007	0.857	0.061	0.735	0.843	0.045	0.700	0.905
	SEAP4	5.897	1.035	0.892	0.065	0.796	0.891	0.023	0.827	0.939
	SEAP5	6.073	0.999	0.838	0.059	0.702	0.828	0.056	0.633	0.894
Perceived info on package	PINF1	5.825	1.113	0.644	0.046	0.415	0.634	0.065	0.472	0.752
	PINF2	5.711	1.113	0.618	0.044	0.381	0.621	0.076	0.462	0.760
	PINF3	4.724	1.421	0.851	0.077	0.725	0.846	0.039	0.769	0.923
	PINF4	5.358	1.317	0.769	0.065	0.591	0.757	0.065	0.564	0.859
Lifestyle	LFST1	4.647	1.786	0.840	0.057	0.706	0.837	0.034	0.749	0.900
	LFST2	4.168	1.779	0.910	0.062	0.829	0.910	0.024	0.859	0.950
Consumer knowledge	CKNW1	4.088	1.514	0.713	0.057	0.508	0.686	0.089	0.419	0.835
	CKNW2	4.190	1.510	0.624	0.050	0.389	0.598	0.107	0.348	0.806
	CKNW3	3.853	1.334	0.900	0.064	0.811	0.906	0.047	0.805	0.997
Perceived value of meat in meals	PMTV1	4.959	1.584	0.769	0.058	0.592	0.767	0.035	0.666	0.825
	PMTV2	3.944	1.659	0.792	0.063	0.627	0.793	0.032	0.681	0.842
	PMTV3	3.377	1.639	0.812	0.064	0.659	0.811	0.030	0.739	0.864
	PMTV4	4.050	1.573	0.734	0.055	0.539	0.740	0.039	0.656	0.807
Consumer choice behaviour	CCB1	4.332	1.521	0.879	0.070	0.773	0.880	0.013	0.846	0.902
	CCB2	3.966	1.367	0.830	0.059	0.689	0.827	0.019	0.769	0.859
	CCB3	3.821	1.503	0.808	0.063	0.653	0.805	0.021	0.746	0.837
	CCB4	4.922	1.335	0.540	0.038	0.292	0.542	0.050	0.447	0.657
Price	PRIC1	4.636	1.412	0.505	0.028	0.164	0.412	0.098	0.209	0.583
	PRIC2	5.220	1.355	0.709	0.061	0.503	0.698	0.070	0.488	0.806
	PRIC3	5.817	1.110	0.907	0.075	0.823	0.901	0.028	0.835	0.948
